# Changing patterns and associated factors of exercise participation and physical activity levels among middle-aged and older adults from 2011-2020 in China

**DOI:** 10.1186/s12966-025-01860-2

**Published:** 2025-11-29

**Authors:** Nan Hua, Xinxia Zhang, Feitong Wu, Yunmei Yang, Qin Zhang, Jing Chen

**Affiliations:** 1 Department of Geriatrics, the First Affiliated Hospital, School of Medicine, Zhejiang University, Hangzhou, 310009 China; 2 Zhejiang Key Laboratory for Diagnosis and Treatment of Physic-chemical and Aging-related Injuries, Hangzhou, 310009 China; 3 Department of Cardiology, the First Affiliated Hospital of USTC, Division of Life Sciences and Medicine, University of Science and Technology of China, Hefei, Anhui 230036 China

**Keywords:** Epidemiology, Exercise participation, Older adults, Physical activity, Health promotion

## Abstract

**Background:**

Physical inactivity remains a global concern. Understanding population-level physical activity (PA) trends is essential for evidence-based policy-making.

**Methods:**

Repeated cross-sectional analysis was conducted using the five waves (2011–2020) of data from the China Health and Retirement Longitudinal Study. PA was assessed by the International Physical Activity Questionnaire. PA sources (job demands, entertainment, exercise, and others) were assessed since 2013. Age- and sex-standardized prevalence was calculated. Log-binomial regression was performed to explore associated factors.

**Results:**

Among Chinese adults ≥ 45 years, weekly exercise participation nearly doubled from 22.6% (2013) to 43.9% (2020). Job-related PA declined but remained the major source of moderate-to-vigorous PA. Insufficient PA prevalence demonstrated slight fluctuations, bottoming at 19.6% in 2018 between 2011 (23.9%) and 2020 (22.2%), with significant reductions in adults ≥ 75 years (-12.7%), females (-5.8%), urban residents (-5.5%), hypertension (-4.1%) or diabetes (-9.7%) groups. By 2020, insufficient PA was more prevalent among adults ≥ 75 years (41%, PR: 2.06, 95% CI: 1.65 to 2.57), individuals with low socioeconomic status (SES) (PR: 1.41, 95% CI: 1.26 to 1.59), rural residents (PR: 1.13, 95% CI: 1.01 to 1.26), and individuals with dyslipidemia (PR:1.12, 95% CI: 1.00 to 1.24).

**Conclusion:**

Our study provides national updated evidence showing exercise participation doubled among middle and older Chinese, yet insufficient PA remained stable over the past decade, which is likely attributable to reductions in job-related PA. In the context of rapid lifestyle and societal changes, future policies and intervention programs need to co-target exercise and varied PA domains and prioritize high-risk populations, including advanced age seniors, rural residents, and low SES groups, to alleviate inequities.

**Supplementary Information:**

The online version contains supplementary material available at 10.1186/s12966-025-01860-2.

## Introduction

Promoting exercise and physical activity (PA) brings benefits not only for the health of individuals, but also for societies and economies [1, 2]. While global efforts have been made to achieve the WHO’s goal of a 15% relative reduction in insufficient PA by 2030 [3], physical inactivity remains prevalent globally [4, 5]. The recent global data on PA revealed that the prevalence of insufficient PA increased to 31% in 2022 among adults. Among older adults, studies showed different trends across countries, with PA engagement remaining stable in the United States [6] but slight declining in Europe [7]. Insufficient PA accounted for 70% of healthcare expenditure on treating illness in high-income countries [8]. Thus, supporting people to be more physically active is a cost-effective strategy for reducing the burden of chronic diseases [9–11], as well as for healthy aging [12].

Over the past decade, population aging has intensified globally and in China. To address the growing burden of non-communicable diseases associated with aging populations, China has implemented a range of measures of health promotion by promoting healthy lifestyles, including exercise [13–15], which might have positive effects on PA engagement among the population. On the other hand, with the rapid development of society and technology, people’s lifestyles tend to be sedentary [16]. Environmental and policies are major drivers of population-level physical activity participation [4]. The trends of PA level among the middle-aged and older population in China remain unclear in the context of rapid societal changes. Moreover, most studies have quantified PA only by duration and intensity, rarely examining its source (e.g., work, exercise) [17, 18]. Understanding this is critical, as modernization may reshape the composition of PA, and focusing solely on total PA may obscure underlying shifts. Examining both overall level and source of PA is therefore essential to evaluate policy impact and inform more targeted health promotion strategies.

By using prospectively collected data from the China Health and Retirement Longitudinal Study (CHARLS) from 2011 to 2020, we aimed to: (1) assess the prevalence and trends in exercise participation and PA levels from 2011 to 2020 among middle-aged and older adults in China; (2) assess the changing trends in the source PA; (3) examine factors associated with insufficient PA and exercise participation, including sociodemographic (age group, sex, living area, socioeconomic status) and health-related factors (presence of hypertension, diabetes, and dyslipidemia).

### Methods

#### Study design and participants

Cross-sectional analyses used data from five CHARLS waves (2011, 2013, 2015, 2018, 2020).The CHARLS was designed to recruit a nationally representative sample of adults aged 45 years and older and has been followed up every 2–3 years since 2011 [19]. The CHARLS adopted a multistage stratified probability-proportionate-to-size sampling strategy covering 150 counties or districts and 450 villages or urban communities (primary sampling units, PSUs) across 28 provinces in China (details in the CHARLS cohort profile [19]). Within each selected PSU, households were randomly sampled. Within each selected household, if one or more members were aged ≥ 40 years, one individual was randomly selected. Selected individuals aged ≥ 45 years were included as main respondents (with their spouses also included), while those aged 40–44 years were considered as a refreshment sample and became eligible for inclusion in subsequent survey waves once they reached 45 years of age. According to the CHARLS cohort profile [19], the demographic characteristics of the participants were comparable to the Chinese census, suggesting the national representativeness of the sample. The CHARLS was approved by the Biomedical Ethics Committee of Peking University, and all the participants provided written informed consent.

A total of 17,708, 18,612, 21097,19816, and 19,385 participants were face-to-face interviewed in each survey wave about demographic information, health behaviors, health outcomes, and others. The current study’s inclusion criterion was participants who responded to the PA survey. The exclusion criterion was participants: (1) aged < 45 years; (2) with missing information on age; (3) with incomplete PA information; (4) without sample weights provided by CHARLS. Participants without sample weights were excluded because CHARLS calculated weights only for the main respondents and their spouses. Individuals without weights were non-sample respondents, such as those mistakenly interviewed [20]. 

### Measurements

#### Physical activity data collection in CHARLS

Physical activity (PA) level in CHARLS were collected using the short form of the International Physical Activity Questionnaire (IPAQ) [21]. In the year 2011, 2013, and 2015 waves, half of the randomly selected participants were invited to complete the PA level assessment, and since 2018, PA level of all CHARLS participants were assessed. Starting from 2013, participants additionally reported the source of their PA.

#### Physical activity levels

The short-form Chinese version of IPAQ-C had good reliability and validity for assessing physical activity level in older Chinese adults [21]. Participants were asked if they engaged in vigorous-, moderate-, and light-intensity PA (such as walking) at least 10 min at a time during a usual week (yes/no), respectively. If participants reported any intensity of PA, frequency (from 1 to 7 days per week), duration of each day (< 30 min, 30 min to 2 h, 2 h to 4 h, and ≥ 4 h) for the corresponding intensity of PA in a week were further asked. We used the median value of each duration range (e.g., 30 min to 2 h was assigned as 75 min) [17]. Thus, the metabolic equivalent (MET) minutes per week to characterize the PA amount were calculated, and the weight of each intensity was according to the IPAQ scoring protocol [22].

Physical activity levels were classified according to the IPAQ scoring protocol as high level of PA (vigorous activity on ≥ 3 days accumulating ≥ 1500 MET-min/week, or any combination of activities on ≥ 7 days accumulating ≥ 3000 MET-min/week), moderate level of PA (≥ 150 min of moderate-intensity activity or ≥ 60 min of vigorous activity per week, or accumulating ≥ 600 MET-min/week), and low level of PA (not meeting the above criteria) [22]. Participants with low level of PA and were defined as having insufficient PA [17, 23].

#### Source of physical activity

From wave 2013, participants reported the primary purpose of their PA (job demand, entertainment, exercise, or others) for each intensity of PA they engaged. Weekly exercise participation was defined as reporting PA with the purpose of exercise per week, regardless of the intensity.

#### Participants characteristics

Sociodemographic characteristics were assessed, including age, sex (male, female), living area (urban, rural), education, and household consumption. Age was divided into four groups: 45–54 years, 55–64 years, 65–74 years, and ≥ 75 years. Education for indicating social status and consumption for indicating economic status were incorporated to measure socioeconomic status (SES). Education level was categorized into three groups: (1) illiterate; (2) elementary school and below; and (3) middle school and above. In CHARLS, household food consumption in the last week, non-food consumption in the last month (e.g., water and electricity fees), and other non-food consumption in the last year (e.g., clothing, travel, and medical expenses) were measured. We calculated per capita annual consumption by multiplying weekly food consumption by 52 weeks, monthly non-food consumption by 12 months, and then adding the annual other non-food expenditure, all divided by the number of household members. We classified per capita annual consumption into four levels based on quartiles [24]. SES score was constructed by combining education level (1, 2, 3) and consumption level (1, 2, 3, 4) [24]. The summed SES score was categorized into three groups: low SES (score 2 or 3), middle SES (score 4 or 5), and high SES (score 6 or 7).

For health-related characteristics, chronic diseases diagnosed by doctors were reported in CHARLS. Hypertension, diabetes, and dyslipidemia are the most common chronic diseases and the focus of chronic disease management in primary care in China [25]. Thus, we selected hypertension, diabetes, and dyslipidemia for analysis.

### Statistical analyses

Due to data availability, we calculated the prevalence of weekly exercise participation and different sources of PA from 2013 to 2020, and the prevalence of PA levels from 2011 to 2020. Subgroup analyses were conducted by age group, sex, living area, SES, the presence of hypertension, diabetes, or dyslipidemia. To ensure comparability across waves, age- and sex-standardized prevalence was calculated using post-stratification weights based on the 2020 China Census population. Trends in prevalence were evaluated through absolute prevalence differences and annual percentage change (APC). APC was derived using log-binomial regression models with a continuous time variable, where APC = 100 × (exp(β) − 1) [26].

Log-binomial regression was used to identify factors associated with exercise participation and insufficient PA based on 2020 data. Multivariable models were adjusted for age group, sex, living area, SES, hypertension, diabetes, and dyslipidemia.

Sample weights accounted for the multi-stage sampling design and non-response bias in CHARLS, ensuring national representativeness. Statistical significance was set at *p* < 0.05 (two-tailed). Analyses were conducted using Stata/MP 18 (StataCorp LLC, College Station, TX, USA).

### Patient and public involvement statement

Patients and the public were not involved in the design, conduct, or dissemination of this study.

## Results

A total of 6593, 5717, 9056, 17,696, and 17,148 participants were included for analyses in each wave (Figure S1). The mean age was 63.7 years (SD 9.7) in the last follow-up in 2020. A slightly higher proportion of participants lived in rural areas (e.g., 62.0% in 2020), and with moderate SES (e.g., 44.4% in 2020) across waves (Table 1).

**Table 1:** Characteristics of study participants (unweighted)

**Characteristics**	2011	2013	2015	2018	2020
	(*n* = 6593)	(*n* = 5717)	(*n* = 9056)	(*n* = 17696)	(*n* = 17148)
Age, mean (SD), y	59.0 (9.6)	59.5 (9.5)	59.8 (9.7)	62.2 (10.0)	63.7 (9.7)
Age group, No. (%)					
45–54 years	2353 (35.7)	1931 (33.8)	3161 (34.9)	4866 (27.5)	3542 (20.1)
55–64 years	2499 (37.9)	2162 (37.8)	3132 (34.6)	5862 (33.1)	5891 (34.4)
65–74 years	1228 (18.6)	1166 (20.4)	1984 (21.9)	4733 (26.8)	5341 (31.2)
≥ 75 years	513 (7.8)	458 (8.0)	779 (8.6)	2235 (12.6)	2464 (14.4)
Sex, No. (%)					
Male	3047 (46.2)	2645 (46.3)	4413 (48.7)	8422 (47.6)	8062 (47.0)
Female	3546 (53.8)	3072 (53.7)	4643 (51.3)	9274 (52.4)	9086 (53.0)
Living area, No. (%)					
Urban	2658 (40.3)	2145 (37.5)	3571 (39.4)	6814 (38.5)	6478 (38.0)
Rural	3935 (59.7)	3572 (62.5)	5485 (60.57)	10,882 (61.5)	10,588 (62.0)
SES, No. (%)					
Low	1748 (27.2)	1476 (26.3)	2332 (26.3)	4736 (27.1)	4464 (26.6)
Moderate	2795 (43.5)	2472 (44.1)	4005 (45.2)	7802 (44.6)	7471 (44.4)
High	1890 (29.4)	1655 (29.6)	2533 (28.6)	4939 (28.3)	4877 (29.0)
Hypertension, No. (%)	1728 (26.3)	1539 (27.2)	2601 (33.4)	6991 (39.5)	7028 (41.0)
Diabetes, No. (%)	400 (6.1)	390 (6.9)	779 (10.1)	2326 (13.1)	2593 (15.1)
Dyslipidemia, No. (%)	672 (10.4)	712 (12.9)	1473 (19.4)	3962 (22.4)	4587 (26.8)

Categorical data were presented as No. (%)

*SES* Socioeconomic status

### The changing trends of the prevalence of weekly exercise participation from 2013 to 2020

**Fig. 1:**
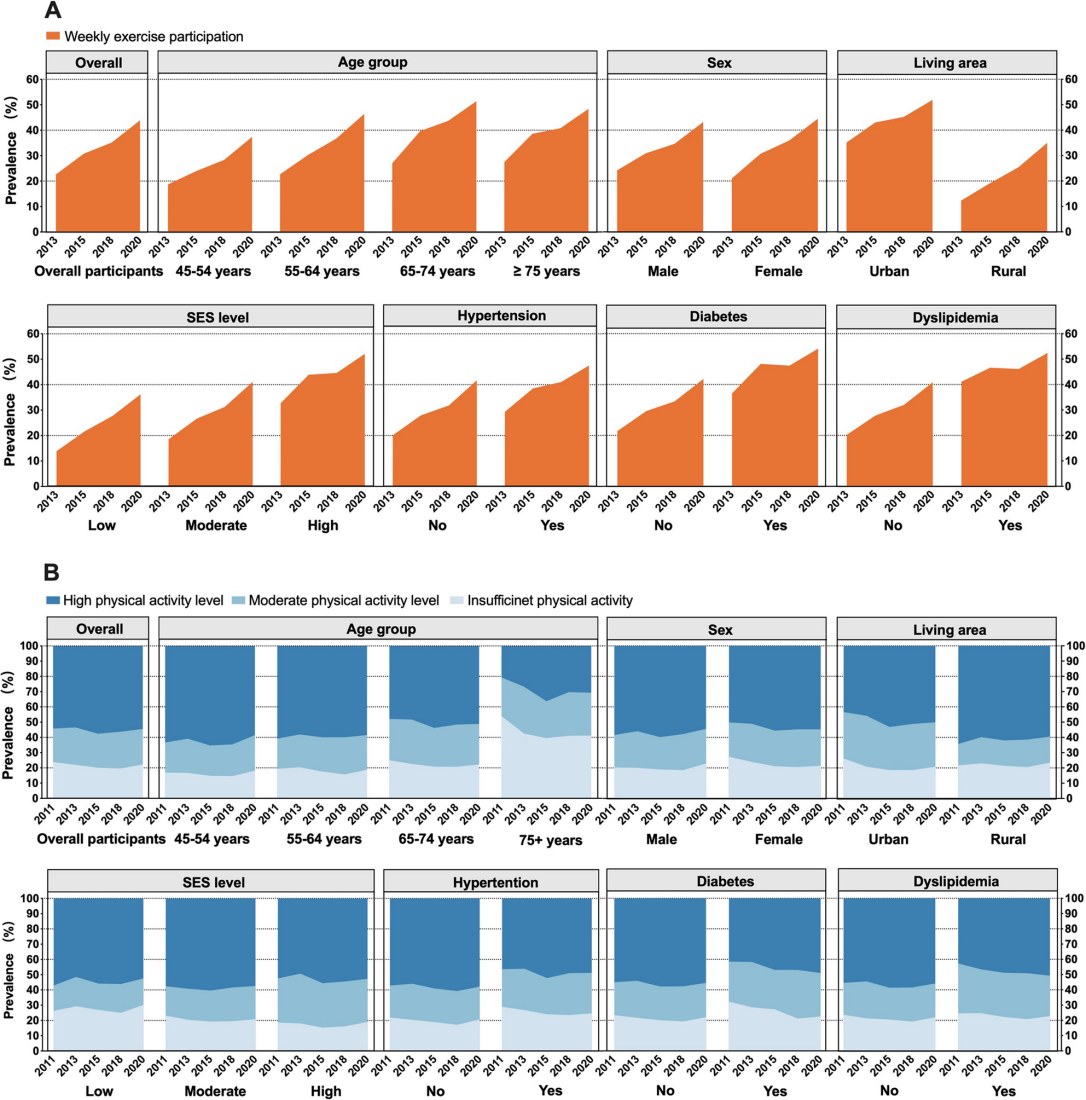
Trends in the age- and sex-standardized prevalence of weekly exercise participation and PA levels (high, moderate, insufficient) among middle-aged and older adults in China by sociodemographic and health status subgroups. **A** Trends in the prevalence of weekly exercise participation from 2013 to 2020; **B** Trends in the prevalence of high PA level, moderate PA level, and insufficient PA from 2011 to 2020. Weekly exercise participation (assessed since 2013 in the CHARLS): defined as reporting physical activity with the purpose of exercise per week, regardless of the intensity. Physical activity level of the study participants was categorized as high level, moderate level, or insufficient according to the International Physical Activity Questionnaire (IPAQ) scoring protocol. SES: socioeconomic status; PA: physical activity

The age- and sex-standardized prevalence of weekly exercise participation increased from 22.6% (95% CI: 19.7 to 25.7) in 2013 to 43.9% (95% CI: 41.8 to 45.9) in 2020, with an absolute increase of 21.3% and an APC of 10.0% among middle-aged and older adults in China (Fig. 1A, Table S1). The greater annual percentage increase in weekly exercise participation was seen among females, rural residents, and individuals with low-to-middle SES (Table S1).

**Fig. 2:**
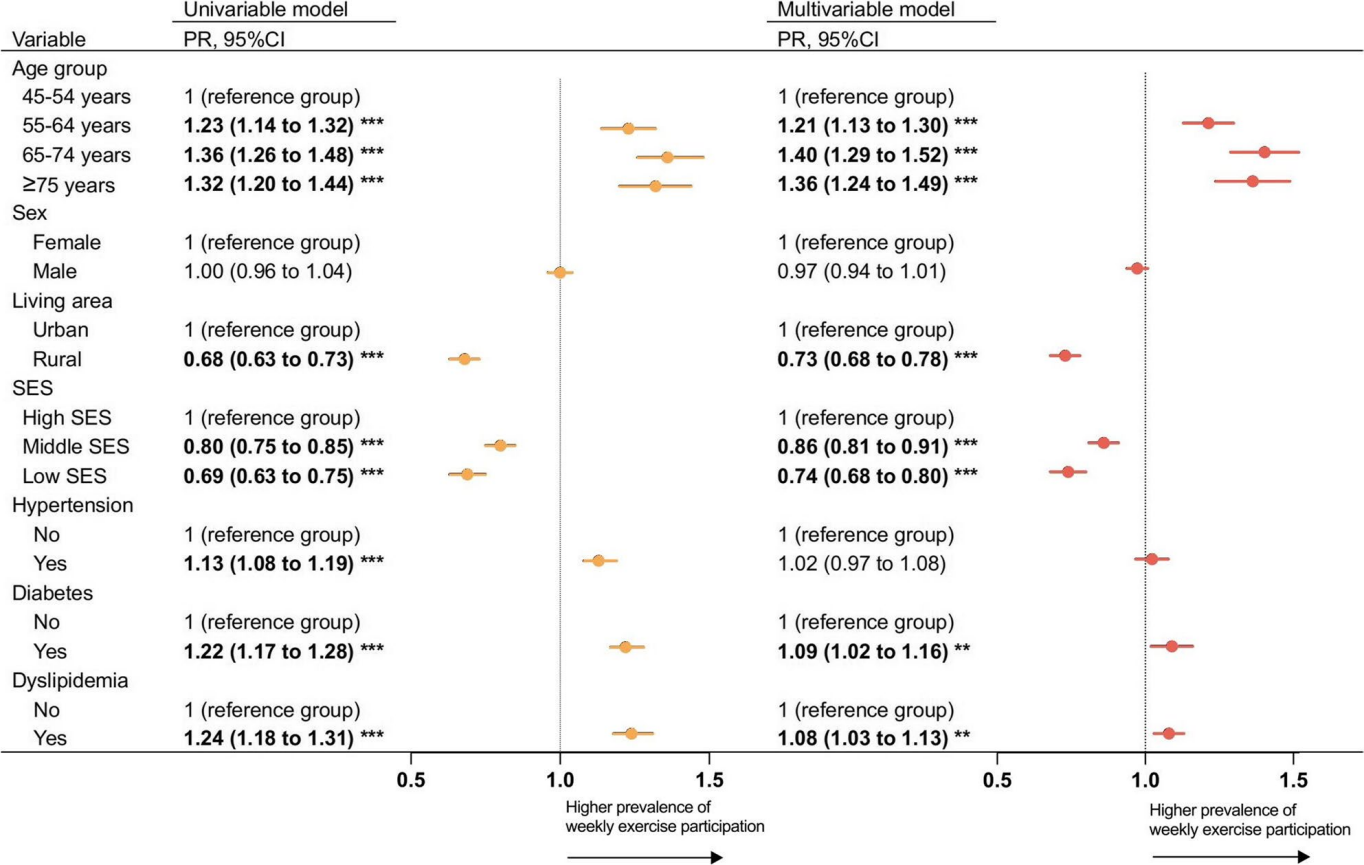
Association between socio-demographic and health factors and prevalence of weekly exercise participation among middle-aged and older adults in 2020 in China. Multivariable model: Age groups, sex, living area, SES, hypertension, diabetes, and dyslipidemia were included in the multivariable model. The direction of the arrow represented a higher prevalence of weekly exercise participation. Data in bold indicated statistically significant. *p < 0.05. **p < 0.01.***p < 0.001. PR: Prevalence ratio. SES: Socioeconomic status

The prevalence of weekly exercise participation was higher in older age groups across waves. In 2020, older adults aged 65–74 years (51.4%) and ≥ 75 years (48.4%) had higher prevalence, compared to 37.4% in those aged 45–54 years (Table S1). The multivariable analysis showed, compared to those aged 45–54 years, the prevalence of weekly exercise participation were1.40 times higher (95% CI: 1.29 to 1.52) among those aged 65–74 years and 1.36 times higher (95% CI 1.24 to 1.49) among those aged ≥ 75 years (Fig. 2).

Despite the increasing trend, the prevalence of weekly exercise participation remained lower among rural residents (35.0%) and those with low SES (36.2%) by 2020. The multivariable analysis showed that compared to the reference group, the prevalence of weekly exercise participation was 0.73 times lower (95% CI: 0.68 to 0.78) among rural residents and 0.74 times lower (95% CI: 0.68 to 0.80) among individuals with low SES (Fig. 2).

The multivariable models showed no significant difference was observed between those with and without hypertension (PR: 1.02, 95% CI: 0.97 to 1.08) and the prevalence of weekly exercise participation were slightly higher among individuals with diabetes (PR 1.09, 95% CI: 1.02 to 1.16) and individuals with dyslipidemia (PR 1.08, 95% CI: 1.03 to 1.13) than those without these conditions (Fig. 2). In 2013, although the prevalence of weekly exercise participation was lower in females than in males (21.0% vs. 24.2%), it became similar between males and females in 2020 (43.2% vs. 44.5%; PR: 0.97, 95% CI: 0.94 to 1.01) (Table S1, Fig. 2).

### Source of PA and changing trends from 2013 to 2020

**Fig. 3:**
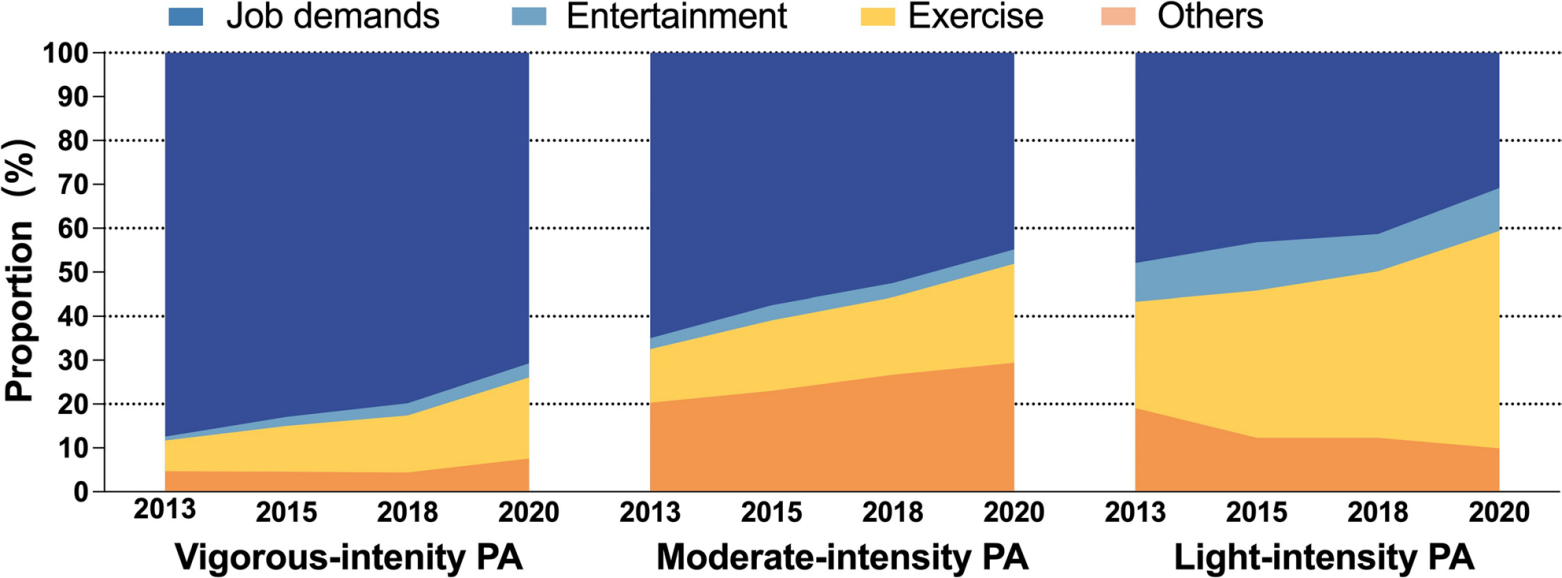
Trends in age- and sex-standardized proportion of different source of PA among middle-aged and older adults from 2013 to 2020 in China, by PA intensity level. Data on PA source was collected since the 2013 wave. The proportions of difference PA source were calculated by dividing the number of participants reporting a specific PA source (e.g., job demands, entertainment, exercise, ethers) by the total number of participants who engaged in any PA of that corresponding intensity level

The composition patterns changed greatly among middle-aged and older Chinese adults from 2013 to 2020 (Fig. 3, Table S2). In 2013, job-related PA was the primary source of PA across all intensity levels and accounted for the highest proportion among vigorous-intensity PA (87.4%). From 2013 to 2020, the proportion of job-related PA decreased but it remained the major source of vigorous-intensity PA (70.7%) and moderate-intensity PA (44.9%).

Meanwhile, the proportion of exercise-related PA increased significantly across different PA intensity levels, with the most notable rise observed in light-intensity PA, increasing from 24.2% in 2013 to 49.5% in 2020 (Fig. 3, Table S2). Exercise superposed job demands, as the leading purpose in light-intensity PA in 2020. By contrast, exercise only accounted for 18.5% of vigorous-intensity PA and 22.5% of moderate-intensity PA in 2020.

### Prevalence of different PA levels and changing trends from 2011 to 2020

The prevalence of PA levels remained stable in middle-aged and older adults in China from 2011 to 2020. Overall, the age- and sex-standardized prevalence of insufficient PA was 23.9% (95% CI: 21.1 to 26.8) in 2011, declined to a low of 19.6% (95% CI: 18.4 to 20.8) in 2018, and rose slightly to 22.2% (95% CI: 20.4 to 24.0) in 2020, with no significant changes observed between 2011 and 2020 (Fig. 1B, Table S3). Among the study population, the overall prevalence of moderate PA level remained stable at around 20% and high PA levels at 55% across waves (Table S4, Table S5).

**Fig. 4:**
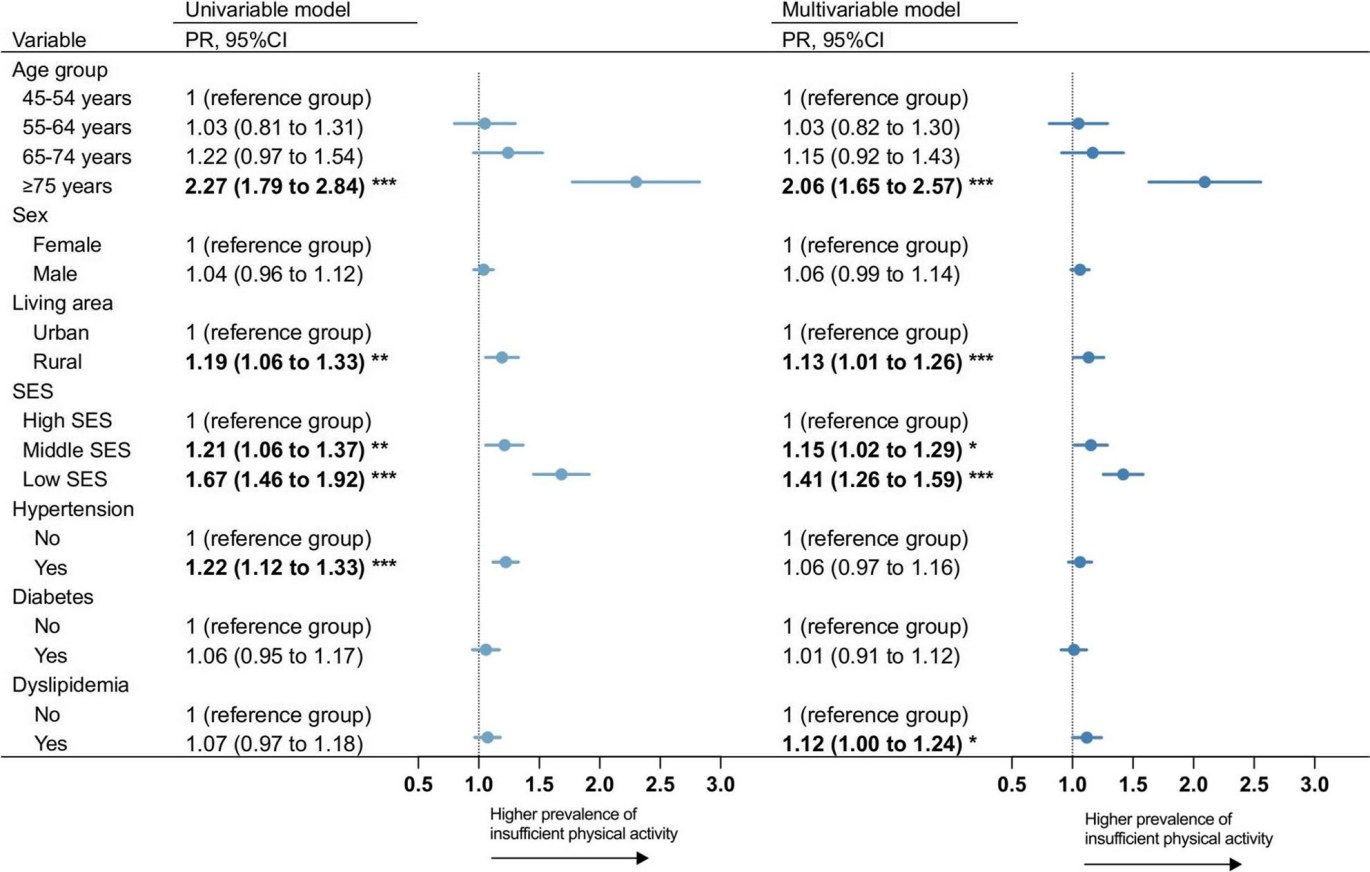
Association between socio-demographic and health factors and prevalence of insufficient physical activity among middle-aged and older adults in 2020 in China. Multivariable model: Age groups, sex, living area, SES, hypertension, diabetes, and dyslipidemia were included in the multivariable models. The direction of the arrow represented a higher prevalence of having insufficient physical activity. Data in bold indicated statistically significant. *p < 0.05. **p < 0.01.***p < 0.001. PR: Prevalence ratio. SES: socioeconomic status

The prevalence of PA levels and changes in trend differed by age groups (Fig. 1B). Despite a 12.7% reduction, 41.2% of adults aged ≥ 75 years still had insufficient PA in 2020, compared to 18.2% among those aged 45–54 years (Table S3). The multivariable analysis showed individuals aged ≥ 75 years had 2.06 times (95% CI: 1.65 to 2.57) higher prevalence of insufficient PA compared to those aged 45–54 years (Fig. 4), but no significant difference was found in other age groups.

With the faster reduction in the prevalence of insufficient PA among urban residents (difference in prevalence: −5.5%, APC: −2.6%) over the years, the prevalence of insufficient PA in urban residents became lower than that in rural residents in 2020 (20.8% vs. 23.5%) (Table S3). Likewise, the gap in the prevalence of high PA level between urban and rural residents was narrowing (Table S5). The multivariable model showed that, compared to urban residents, rural residents had a 1.13 times higher prevalence of insufficient PA in 2020 (Fig. 4).

Individuals with low SES had the highest prevalence of insufficient PA across waves, and the gap in the prevalence between high and low SES appeared to be widening (Table S3). The multivariable analysis showed that the prevalence of insufficient PA was 1.41 times (95% CI: 1.26 to 1.59) higher among the group with low SES compared to high SES in 2020 (Fig. 4).

During the study period, significant reductions in insufficient PA prevalence were observed in those with hypertension (difference in prevalence: −4.1%, APC: −1.7%) or diabetes (difference in prevalence: −9.7%, APC: −3.9%) (Table S3). In 2020, the multivariable model showed that no difference in the prevalence of insufficient PA between people with hypertension, diabetes, and without these conditions, but the prevalence was 1.12 times higher among people with dyslipidemia (95% CI: 1.00 to 1.24) than those without dyslipidemia (Fig. 4). With the faster reduction in the prevalence of insufficient PA among females (difference in prevalence: −5.8%, APC: −2.6%), the prevalence of insufficient PA became similar between females and males (21.4% vs. 22.9%) in 2020, with an PR of 1.06 (95% CI: 0.99 to 1.14) (Table S3, Fig. 4).

## Discussion

Using nationally representative data, our study provided the latest comprehensive analysis of exercise and PA trends among Chinese adults aged ≥ 45 over the past decade (2011–2020). The prevalence of weekly exercise participation increased from 22.6% (2013) to 43.9% (2020). Meanwhile, job-related PA declined but remained the main moderate-to-vigorous PA source. Despite increased exercise participation, insufficient PA prevalence remained stable from 23.9% (2011) to 22.2% (2020), reaching its lowest point of 19.6% in 2018. Significant declines in the prevalence were observed among adults ≥ 75 years (−12.7%), females (−5.8%), urban residents (−5.5%), and those with hypertension (−4.1%) or diabetes (−9.7%) during the study period. By 2020, Insufficient PA was prevalent among those aged ≥ 75 years (41%), rural residents, individuals with lower SES, and dyslipidemia. Our findings highlight the importance of further targeted policies and interventions to promote intentional exercise and varied PA domains in daily routines, especially for advanced age seniors, rural residents, and individuals with low SES, to address inequities.

Our study demonstrated a significant decadal rise in exercise participation among China’s middle-aged and older adults (22.6% in 2013 to 43.9% in 2020), suggesting the possible efficacy of targeted public health policies. Guided by the “National Fitness Program” [15] launched in 2011 and the “Healthy China 2030” [14] launched in 2016, the government subsidizes exercise infrastructure at communities, scales up public health education, and lifestyle counseling for individuals with chronic conditions. However, the findings also showed that the prevalence of hypertension, diabetes, and dyslipidemia has increased, which was consistent with other studies [27]. While the specific reasons for this increase remains unclear, insufficient exercise volume and intensity, unhealthy dietary habits, and strengthened disease detection in primary care my partly explained the observed trend [27]. These findings suggested that to better control modifiable risk factors for chronic diseases in the population, implementation of comprehensive strategies that integrate healthy diet, smoking cessation, alcohol control, and weight management into sustained practice is needed.

On the other hand, rising exercise engagement did not lead to significant population-level PA improvements, likely due to a concurrent decline in occupational PA. During the study period, China’s industrial structure has transitioned from primary and secondary industries to tertiary industries [28], with physically demanding jobs reducing. This aligned with our results of a persistently declining proportion of job-related PA. These findings uncovered a crucial public health challenge in mitigating the decline of PA domains related to modernization while promoting intentional exercise. Additionally, we observed differences by age group, sex, living areas and SES in the trends of PA levels and exercise participation within the context of social transitions and evolving health policies. These factors may even interact with each other, warranting further exploration to inform more targeted health promotion strategies.

We identified PA disparities across sub-populations with significantly higher prevalence of insufficient PA among adults of advanced age, rural residents, and individuals with low SES. These high-risk populations face barriers that cause such inequities in PA. At the individual level, they tend to have lower health literacy, limited awareness of the importance of PA and exercise [29–31]. At the environmental level, rural and lower SES areas generally lack facilities and resources for leisure-time PA (such as gyms and cycling lanes) [32–34]. In particular, insufficient PA was most prevalent among advanced-age adults (41%), nearly two times higher than that of younger age groups. Retirement may partly account for the observed difference, as retired older adults experienced a decline in work-related PA [35]. Older adults may have more leisure time to engage in exercise, which is consistent with our findings that exercise participation was higher among older adults than middle-aged adults. According to WHO and other PA guidelines, the recommended PA level and intensity for older adults was not lower than that for younger adults [36, 37]. These findings suggested that promoting public education on exercise and PA engagement, with an emphasis on achieving sufficient intensity and duration, remains essential to reduce the heavy burden of insufficient PA and to achieve equitable healthy aging.

The main strength of this study is the use of the most recent nationally representative CHARLS data. We analyzed both PA levels and sources, including weekly exercise participation, offering a detailed view of PA among middle-aged and older adults in China. However, our study has limitations. Firstly, PA was assessed by questionnaire, not objectively measured. The data was collected via face-to-face interviews using the validated IPAQ short form by trained staff [38], and this approach is widely used in large-scale PA surveillance [23]. Secondly, the CHARLS adopted categorical responses instead of the continuous responses in the original IPAQ questions about PA duration, which might have caused measurement errors. Previous CHARLS studies have consistently showed the associations between low PA level and poor health outcomes [39, 40], supporting its reasonable performance assessing PA level in the study population. Thirdly, while classifying PA by source was widely used in PA questionnaires [41, 42], the questions assessing sources of PA in the CHARLS have not been formally validated. Additionally, PA purpose was identified through a single-choice question (job, entertainment, exercise, or others) to indicate the primary source. Since PA of different intensities can have multiple sources, this may underestimate certain types like exercise, though it has little influence on trends and subgroup comparisons.

## Conclusion

Our study provides novel updated national evidence on the evolving exercise participation and PA landscape among middle-aged and older Chinese adults over the recent decade (2011–2020). Critically, exercise participation almost doubled (from 22.6% to 43.9%), suggesting a promising shift towards proactive health engagement in the study population. However insufficient PA prevalence remained paradoxically stable which is likely attributable to reductions in job-related PA, underscoring the complex interplay between lifestyle choices and broader societal shifts. Significant reductions in insufficient PA prevalence were observed in adults ≥ 75 years, females, urban residents, and individuals with hypertension or diabetes. By 2020, insufficient PA was most prevalent among adults aged ≥ 75 years (41%, two times higher than younger age groups), rural residents, and those with low SES or dyslipidemia. These findings necessitate precision interventions co-targeting intentional exercise and varied PA domains in daily routines and prioritizing high-risk populations to promote overall PA levels and alleviate inequities among populations.

## Supplementary Information

Supplementary Material 1

Supplementary Material 2

## Data Availability

The data used in the study are accessible to be downloaded publicly at (http://charls.pku.edu.cn).

## Abbreviations

APC: Annual Percentage Change
CHARLS: China Health and Retirement Longitudinal Study
MET: Metabolic Equivalent
PA: Physical Activity
SES: Socioeconomic Status
WHO: World Health Organization
