# Supplementary material for: Changing patterns and associated factors of exercise participation and physical activity levels among middle-aged and older adults from 2011-2020 in China
Source: Int J Behav Nutr Phys Act. 2025 Nov 29;23:2. doi: 10.1186/s12966-025-01860-2 (PMC12781555; doi:10.1186/s12966-025-01860-2)
Supplement: Supplementary file 1 — Supplementary Material 1 [file 12966_2025_1860_MOESM1_ESM.docx]

*Supplementary Content*

**Table S1.** Age- and sex-standardized prevalence, difference and annual percentage change in prevalence of weekly exercise participation among middle and older-aged adults from 2013 to 2020 in China.

**Table S2.** Proportion and difference in proportion of purposes for light, moderate, vigorous intensity physical activity among middle and older-aged adults from 2013 to 2020 in China.

**Table S3.** Age- and sex-standardized prevalence, difference and annual percentage change in prevalence of insufficient physical activity level among middle-aged and older adults from 2011 to 2020 in China.

**Table S4.** Age- and sex-standardized prevalence, difference in prevalence of moderate physical activity level among middle and older-aged adults from 2011 to 2020 in China.

**Table S5.** Age- and sex-standardized prevalence, difference in prevalence of high

physical activity level among middle and older-aged adults from 2011 to 2020 in China.

**Figure S1.** Flow diagram of study design.

This supplementary material has been provided by the authors to give readers additional information about their work.

| **Table S1.** Age- and sex-standardized prevalence, difference and annual percentage change in prevalence of weekly exercise participation among middle and older-aged adults from 2013 to 2020 in China. | | | | | | |
| --- | --- | --- | --- | --- | --- | --- |
|  | Prevalence (95% CI) | | | | Difference in prevalence (95% CI) | APC in prevalence  (95% CI） |
| **Characteristic** | 2013 | 2015 | 2018 | 2020 | 2013-2020 | 2013-2020 |
| *Overall participants* | 22.6 (19.7 to 25.7) | 30.8 (27.6 to 34.1) | 35.2 (33.1 to 37.4) | 43.9 (41.8 to 45.9) | **21.3 (18.9 to 23.7) ***** | **10.0 (8.24 to 11.7) ***** |
| *Age group* |  |  |  |  |  |  |
| 45-54 years | 18.7 (15.5 to 22.4) | 23.9 (21.3 to 26.7) | 28.3 (25.9 to 30.8) | 37.4 (34.7 to 40.2) | **18.7 (14.6 to 22.9) ***** | **10.4 (7.4 to 13.4) ***** |
| 55-64 years | 22.7 (19.3 to 26.5) | 30.3 (26.8 to 34.1) | 36.7 (33.4 to 40.0) | 46.4 (43.7 to 49.0) | **23.6 (20.4 to 26.8) ***** | **10.7 (8.6 to 12.9) ***** |
| 65-74 years | 27.0 (22.8 to 31.6) | 39.7 (36.2 to 43.4) | 43.7 (40.5 to 47.0) | 51.4 (48.5 to 54.3) | **24.4 (20.1 to 28.7) ***** | **9.6 (7.3 to 12.0) ***** |
| ≥75 years | 27.5 (22.0 to 33.7) | 38.6 (28.1 to 50.2) | 40.8 (37.9 to 43.9) | 48.4 (45.1 to 51.8) | **21.0 (15.6 to 26.3) ***** | **8.4 (5.5 to 11.4) ***** |
| *Sex* |  |  |  |  |  |  |
| Male | 24.2 (21.1 to 27.7) | 30.9 (28.1 to 33.8) | 34.6 (32.2 to 37.1) | 43.2 (40.8 to 45.6) | **19.0 (15.9 to 22.0) ***** | **8.6 (6.7 to 10.5) ***** |
| Female | 21.0 (26.8 to 34.9) | 30.7 (26.8 to 34.9) | 35.9 (33.6 to 38.3) | 44.5 (42.4 to 46.7) | **23.6 (20.7 to 26.4) ***** | **11.4 (9.1 to 13.6) ***** |
| *Living area* |  |  |  |  |  |  |
| Urban | 35.1 (30.0 to 40.7) | 43.0 (37.8 to 48.3) | 45.2 (42.2 to 48.2) | 51.9 (49.1 to 54.7) | **16.8 (12.8 to 20.8) ***** | **5.7 (3.9 to 7.6) ***** |
| Rural | 12.3 (10.4 to 14.5) | 19.1 (17.5 to 20.8) | 25.4 (23.6 to 27.3) | 35.0 (33.0 to 37.1) | **22.7 (20.5 to 25.0) ***** | **16.1 (13.6 to 18.6) ***** |
| *SES* |  |  |  |  |  |  |
| Low SES | 13.8 (11.4 to 16.7) | 21.6 (19.2 to 24.1) | 27.7 (25.5 to 29.9) | 36.2 (33.1 to 39.4) | **22.4 (18.6 to 26.1) ***** | **14.7 (11.5 to 17.9) ***** |
| Middle SES | 18.5 (16.1 to 21.3) | 26.6 (22.1 to 31.6) | 31.2 (29.2 to 33.3) | 41.1 (38.6 to 43.6) | **22.5 (19.8 to 25.2) ***** | **12.0 (10.0 to 14.0) ***** |
| High SES | 32.7 (27.8 to 38.0) | 43.9 (40.0 to 47.9) | 44.6 (41.4 to 47.8) | 52.1 (49.3 to 54.9) | **19.4 (15.6 to 23.2) ***** | **6.9 (5.0 to 8.8) ***** |
| *Hypertension* |  |  |  |  |  |  |
| No | 20.2 (17.4 to 23.5) | 27.9 (25.2 to 30.8) | 31.8 (29.7 to 34.0) | 41.7 (39.5 to 44.1) | **21.5 (18.4 to 24.5) ***** | **10.9 (8.7 to 13.0) ***** |
| Yes | 29.3 (25.1 to 33.9) | 38.5 (32.9 to 44.3) | 41.0 (38.1 to 43.9) | 47.5 (44.9 to 50.2) | **18.2 (14.4 to 22.0) ***** | **7.1 (5.2 to 9.1) ***** |
| *Diabetes* |  |  |  |  |  |  |
| No | 21.7 (18.9 to 24.8) | 29.5 (26.2 to 33.0) | 33.4 (31.4 to 35.6) | 42.2 (40.2 to 44.2) | **20.5 (18.0 to 23.0) ***** | **10.0 (8.2 to 11.8) ***** |
| Yes | 36.5 (29.9 to 43.6) | 48.1 (42.4 to 53.9) | 47.4 (43.1 to 51.7) | 54.1 (40.8 to 57.3) | **17.6 (10.9 to 24.3) ***** | **5.8 (3.1 to 8.5) ***** |
| *Dyslipidemia* |  |  |  |  |  |  |
| No | 20.2 (17.6 to 23.1) | 27.8 (24.3 to 31.6) | 32.0 (30.0 to 34.1) | 40.9 (38.8 to 42.9) | **20.7 (18.1 to 23.3) ***** | **10.6 (8.7 to 12.5) ***** |
| Yes | 41.1 (35.7 to 47.3) | 46.6 (42.0 to 51.1) | 46.1 (43.4 to 48.8) | 52.4 (49.5 to 55.4) | **11.0 (6.0 to 16.1) ***** | **3.4 (1.6 to 5.2) ***** |
| Data in bold indicate statistically significant. **p < 0.01.***p < 0.001.  APC: Annual percentage change. SES: socioeconomic status. | | | | | | |

| Table S2. Proportion and difference in proportion of purposes for light, moderate, vigorous intensity physical activity among middle and older-aged adults from 2013 to 2020 in China. | | | |
| --- | --- | --- | --- |
|  | Prevalence (95% CI) | | |
|  | Light-intensity physical activity | Moderate-intensity physical activity | Vigorous-intensity physical activity |
| **Purposes for physical activity** |  |  |  |
| *Job demands* |  |  |  |
| 2013 | 48.0 (44.0 to 52.0) | 65.0 (60.6 to 69.2) | 87.4 (84.2 to 89.9) |
| 2015 | 43.3 (39.8 to 46.8) | 57.4 (54.2 to 60.6) | 82.9 (80.1 to 85.4) |
| 2018 | 41.2 (38.8 to 43.7) | 52.4 (49.1 to 55.8) | 79.8 (75.6 to 83.4) |
| 2020 | 30.8 (28.3 to 33.4) | 44.9 (42.0 to 47.9) | 70.7 (66.4 to 74.6) |
| Difference in proportion between 2013-2020 (95% CI) | **-17.2 (-20.7 to -13.7) ***** | **-20.1 (-24.3 to -15.8) ***** | **-16.7 (-21.2 to -12.1) ***** |
| *Entertainment* |  |  |  |
| 2013 | 8.8 (7.5 to 10.2) | 2.5 (1.7 to 3.5) | 0.9 (0.5 to 1.7) |
| 2015 | 11.0 (9.6 to 12.4) | 3.4 (2.7 to 4.3) | 2.1 (1.3 to 3.3) |
| 2018 | 8.5 (7.3 to 9.9) | 3.2 (2.5 to 4.1) | 2.8 (1.5 to 5.1) |
| 2020 | 9.8 (8.6 to 11.1) | 3.3 (2.8 to 3.8) | 3.2 (2.1 to 4.9) |
| Difference in proportion between 2013-2020 (95% CI) | 1.0 (-0.7 to 2.7) | 0.8 (-0.2 to 1.8) | **2.3 (0.8 to 3.7) **** |
| *Exercise* |  |  |  |
| 2013 | 24.2 (21.0 to 27.7) | 12.2 (10.1 to 14.7) | 7.0 (5.1 to 9.5) |
| 2015 | 33.5 (30.0 to 37.2) | 16.1 (14.2 to 18.3) | 10.4 (8.6 to 12.5) |
| 2018 | 37.9 (35.6 to 40.3) | 17.6 (15.7 to 19.7) | 13.0 (10.7 to 15.8) |
| 2020 | 49.5 (47.4 to 51.6) | 22.5 (20.4 to 24.7) | 18.5 (16.4 to 20.9) |
| Difference in proportion between 2013-2020 (95% CI) | **25.3 (22.3 to 28.3) ***** | **10.2 (7.7 to 12.7) ***** | **11.5 (9.3 to 13.8) ***** |
| *Others* |  |  |  |
| 2013 | 19.1 (15.7 to 23.0) | 20.3 (17.7 to 23.1) | 4.7 (3.6 to 6.2) |
| 2015 | 12.3 (10.2 to 14.6) | 23.0 (20.9 to 25.2) | 4.6 (3.7 to 5.9) |
| 2018 | 12.3 (11.1 to 13.6) | 26.7 (24.5 to 29.0) | 4.4 (3.6 to 5.4) |
| 2020 | 9.9 (8.7 to 11.3) | 29.4 (26.7 to 32.1) | 7.6 (5.8 to 9.8) |
| Difference in proportion between 2013-2020 (95% CI) | **-9.1 (-12.2 to -6.1) ***** | **9.1 (5.6 to 12.5) ***** | **2.9 (0.4 to 5.4) *** |
| Data in bold indicate statistically significant; *p < 0.05. **p < 0.01.***p < 0.001. | | | |

| **Table S3.** Age- and sex-standardized prevalence, difference and annual percentage change in prevalence of insufficient physical activity level among middle-aged and older adults from 2011 to 2020 in China. | | | | |
| --- | --- | --- | --- | --- |
|  | Prevalence  (95% CI) | Prevalence  (95% CI) | Difference in prevalence (95% CI） | APC in prevalence (95% CI） |
| **Characteristics** | 2011 | 2020 | 2011-2020 | 2011-2020 |
| *Overall* | 23.9 (21.1 to 268) | 22.2 (20.4 to 24.0) | -1.7 (-5.1 to 1.7) | -0.8 (-2.4 to 0.8) |
| *Age group* |  |  |  |  |
| 45-54 years | 16.8 (13.4 to 20.8) | 18.2 (14.6 to 22.5) | 1.5 (-4.3 to 7.2) | 0.9 (-2.7 to 4.6) |
| 55-64 years | 19.4 (15.7 to 23.7) | 18.8 (17.0 to 20.7) | -0.6 (-4.3 to 3.2) | -0.3 (-2.5 to 1.8) |
| 65-74 years | 25.0 (21.7 to 28.7) | 22.3 (20.3 to 24.5) | -2.7 ( -6.4 to 0.9) | -1.3 (-2.9 to 0.4) |
| ≥75 years | 53.9 (47.6 to 60.1) | 41.2 (38.2 to 44.2) | **-12.7 (-19.3 to -0.5) ***** | **-2.9 (-4.3 to -1.6) **** |
| *Sex* |  |  |  |  |
| Male | 20.3 (17.6 to 23.2) | 22.9 (20.7 to 25.3) | 2.7 (-0.9 to 6.2) | 1.4 (-0.5 to 3.2) |
| Female | 27.3 (23.7 to 31.1) | 21.4 (19.6 to 23.3) | **-5.8 (-3.4 to -1.2) **** | **-2.6 (-4.6 to -0.7) **** |
| *Living area* |  |  |  |  |
| Urban | 26.2 (21.9 to 31.1) | 20.8 (18.2 to 23.7) | **-5.5 (-10.8 to -0.2) *** | **-2.6 (-4.9 to -0.2) *** |
| Rural | 21.7 (18.9 to 24.6) | 23.5 (21.5 to 25.6) | 1.9 (-1.7 to 5.4) | 0.9 (-0.8 to 2.7) |
| *SES* |  |  |  |  |
| Low | 26.3 (22.5 to 30.4) | 30.2 (27.2 to 33.4) | 4.0 (-0.7 to 8.7) | 1.6 (-0.3 to 3.5) |
| Middle | 23.2 (20.1 to 26.7) | 20.8 (19.3 to 22.3) | -2.4 (-6.2 to 1.4) | -1.2 (-3.0 to 0.6) |
| High | 18.7 (15.8 to 22.0) | 19.1 (15.7 to 22.9) | 0.4 (-4.4 to 5.1) | 0.2 (-2.6 to 3.0) |
| *Hypertension* |  |  |  |  |
| No | 21.9 (18.9 to 25.2) | 20.7 (18.6 to 23.0) | 1.2 (-5.1 to 2.7) | -0.6 (-2.6 to 1.4) |
| Yes | 28.9 (25.4 to 32.7) | 24.7 (22.9 to 26.6) | **-4.1 (-8.3 to -0.0)** * | **-1.7 (-3.3 to -0.1) *** |
| *Diabetes* |  |  |  |  |
| No | 23.4 (20.6 to 26.5) | 22.1 (20.2 to 24.2) | -1.3 (-5.0 to 2.3) | -0.7 (-2.4 to 1.1) |
| Yes | 32.3 (25.6 to 39.7) | 22.6 (20.3 to 25.0) | **-9.7 (-16.9 to -2.5) **** | **-3.9 (-6.4 to -1.4) **** |
| *Dyslipidemia* |  |  |  |  |
| No | 23.7 (21.1 to 26.5) | 22.0 (19.9 to 24.1) | -1.7 (-5.5 to 2.0) | -0.8 (-2.6 to 0.9) |
| Yes | 24.6 (18.5 to 31.8) | 22.8 (20.6 to 25.1) | -1.8 (-7.9 to 4.3) | -0.8 (-3.6 to 1.9) |
| Data in bold indicated statistically significant. *p < 0.05. **p < 0.01.***p < 0.001.  APC: Annual percentage change. SES: Socioeconomic status. | | | | |

| **Table S4.** Age- and sex-standardized prevalence, difference in prevalence of moderate physical activity level among middle and older-aged adults from 2011 to 2020 in China. | | | |
| --- | --- | --- | --- |
|  | Prevalence (95% CI) | Prevalence (95% CI) | Difference in prevalence (95% CI） |
| **Characteristics** | 2011 | 2020 | 2011-2020 |
| *Overall* | 21.9 (19.6 to 24.5) | 23.3 (21.7 to 25.1) | 1.4 (-0.8 to 3.6) |
| *Age group* |  |  |  |
| 45-54 | 19.8 (16.8 to 23.2) | 20.8 (18.1 to 23.7) | 1.0 (-3.3 to 5.4) |
| 55-64 | 19.8 (16.8 to 23.1) | 22.7 (21.0 to 24.6) | **3.0 (0.1 to 5.8) *** |
| 65-74 | 27.1 (23.4 to 31.0) | 26.5 (23.9 to 29.1) | -0.6 (-4.3 to 3.1) |
| 75+ | 25.4 (21.2 to 30.1) | 28.1 (24.3 to 32.2) | 2.7 (-2.7 to 8.2) |
| *Sex* |  |  |  |
| Male | 21.3 (18.4 to 24.5) | 22.7 (20.6 to 25.1) | 1.4 (-2.2 to 5.1) |
| Female | 22.5 (19.7 to 25.7) | 23.9 (22.3 to 25.7) | 1.4 (-1.4 to 4.2) |
| *Living area* |  |  |  |
| Urban | 30.4 (26.7 to 34.5) | 29.1 (26.1 to 32.3) | -1.3 (-5.4 to 2.7) |
| Rural | 14.0 (12.5 to 15.7) | 17.0 (15.8 to 18.4) | **3.0 (1.3 to 4.8) ***** |
| *SES level* |  |  |  |
| Low | 16.6 (14.3 to 19.3) | 17.3 (15.6 to 19.1) | 0.7 (-2.2 to 3.5) |
| Middle | 19.2 (16.8 to 21.8) | 21.8 (20.0 to 23.8) | **2.6 (0.2 to 5.1) *** |
| High | 28.8 (25.0 to 32.8) | 28.2 (24.6 to 32.0) | -0.6 (-5.5 to 4.3) |
| *Hypertension* |  |  |  |
| No | 21.0 (18.6 to 23.6) | 21.5 (19.4 to 23.7) | 0.5 (-2.6 to 3.6) |
| Yes | 24.7 (24.4 to 30.0) | 26.5 (24.5 to 28.7) | 1.9 (-2.1 to 5.8) |
| *Diabetes* |  |  |  |
| No | 21.6 (19.3 to 24.2) | 22.5 (20.9 to 24.2) | 0.9 (-1.5 to 3.2) |
| Yes | 26.2 (21.2 to 31.8) | 28.4 (25.7 to 31.2) | 2.2 (-3.1 to 7.6) |
| *Dyslipidemia* |  |  |  |
| No | 20.9 (18.6 to 23.3) | 22.2 (20.4 to 24.1) | 1.3 (-1.2 to 3.9) |
| Yes | 32.7 (27.3 to 38.5) | 26.5 (24.5 to 28.7) | **-6.1 (-11.5 to -0.7) *** |
| Data in bold indicate statistically significant; *p < 0.05. **p < 0.01.***p < 0.001.  SES: Socioeconomic status. | | | |

| **Table S5.** Age- and sex-standardized prevalence, difference in prevalence of high physical activity level among middle and older-aged adults from 2011 to 2020 in China. | | | |
| --- | --- | --- | --- |
|  | Prevalence (95% CI) | Prevalence (95% CI) | Difference in prevalence (95% CI） |
| **Characteristics** | 2011 | 2020 | 2011-2020 |
| *Overall* | 54.2 (50.7 to 57.6) | 54.5 (52.4 to 56.7) | 0.3 (-3.3 to 3.9) |
| *Age group* |  |  |  |
| 45-54 | 63.4 (58.4 to 68.2) | 61.0 (56.7 to 65.1) | -2.5 (-9.4 to 4.4) |
| 55-64 | 60.9 (56.3 to 65.3) | 58.5 (56.2 to 60.7) | -2.4 (-6.7 to 1.9) |
| 65-74 | 47.9 (43.3 to 52.6) | 51.3 (48.5 to 54.0) | 3.3 (-1.2 to 7.9) |
| 75+ | 20.8 (16.9 to 25.2) | 30.7 (27.5 to 34.2) | **10.0 (4.7 to 15.3) ***** |
| *Sex* |  |  |  |
| Male | 58.5 (54.4 to 62.4) | 54.4 (51.8 to 56.8) | **-4.1 (-8.2 to -0.0) *** |
| Female | 50.2 (46.6 to 53.8) | 54.7 (52.4 to 56.9) | **4.4 (0.3 to 8.6) *** |
| *Living area* |  |  |  |
| Urban | 43.3 (38.7 to 48.0) | 50.1 (47.0 to 53.3) | **6.8 (1.3 to 12.3) *** |
| Rural | 64.3 (61.1 to 67.5) | 59.4 (56.7 to 62.1) | **-4.9 (-8.7 to -1.0) *** |
| *SES level* |  |  |  |
| Low | 57.1 (53.0 to 61.1) | 52.5 (49.0 to 56.0) | -4.6 (-9.5 to 0.2) |
| Middle | 57.6 (53.6 to 61.5) | 57.4 (54.8 to 59.9) | -0.2 (-4.4 to 4.0) |
| High | 52.5 (48.2 to 43.4) | 52.8 (48.8 to 56.7) | 0.3 (-5.9 to 6.4) |
| *Hypertension* |  |  |  |
| No | 57.1 (53.2 to 60.9) | 57.8 (55.1 to 60.4) | 0.7 (-4.0 to 5.5) |
| Yes | 46.5 (41.9 to 51.0) | 48.7 (46.5 to 51.0) | 2.3 (-2.4 to 7.0) |
| *Diabetes* |  |  |  |
| No | 54.9 (51.4 to 58.4) | 55.4 (53.1 to 57.6) | 4.9 (-3.3 to 4.3) |
| Yes | 41.6 (34.3 to 49.2) | 49.0 (45.8 to 52.3) | 7.5 (-0.2 to 15.1) |
| *Dyslipidemia* |  |  |  |
| No | 55.4 (52.0 to 58.8) | 55.8 (53.4 to 58.3) | 0.4 (-3.6 to 4.4) |
| Yes | 42.8 (37.0 to 48.8) | 50.7 (48.0 to 53.3) | **7.9 (2.3 to 13.5) **** |
| Data in bold indicate statistically significant; *p < 0.05. **p < 0.01.***p < 0.001.  SES: socioeconomic status. | | | |


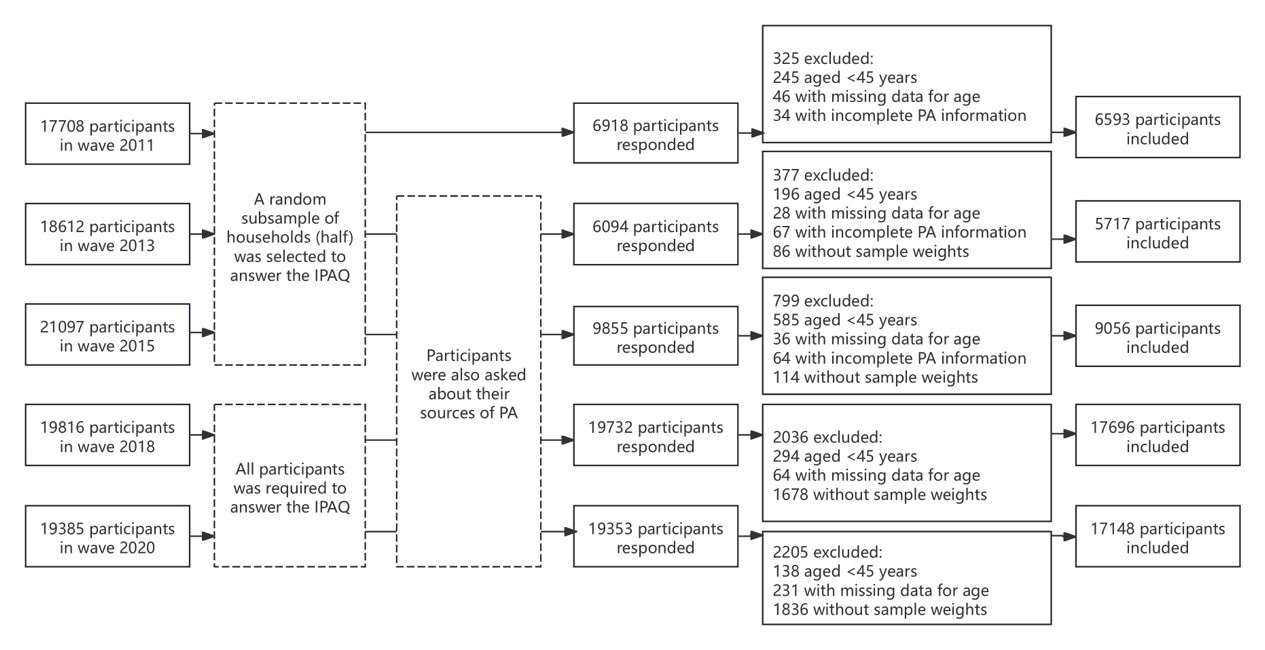


**Figure S1.** Flow diagram of study design.

*The participants included in wave 2011 were re-visited in each follow-up (wave 2013, wave 2015, wave 2018, wave 2020), and new participants were also ongoing recruited in CHARLS to better reflect the representativeness of the population.

IPAQ: the International Physical Activity Questionnaire; PA: Physical activity.
